# Suitability of GRK Antibodies for Individual Detection and Quantification of GRK Isoforms in Western Blots

**DOI:** 10.3390/ijms23031195

**Published:** 2022-01-21

**Authors:** Mona Reichel, Verena Weitzel, Laura Klement, Carsten Hoffmann, Julia Drube

**Affiliations:** Institut für Molekulare Zellbiologie, CMB—Center for Molecular Biomedicine, Universitätsklinikum Jena, Friedrich-Schiller-Universität Jena, Hans-Knöll-Straße 2, D-07745 Jena, Germany; Mona.Reichel@med.uni-jena.de (M.R.); Verena.Weitzel@med.uni-jena.de (V.W.); Laura.Klement@med.uni-jena.de (L.K.); Carsten.Hoffmann@med.uni-jena.de (C.H.)

**Keywords:** GRK2, GRK3, GRK5, GRK6, antibody specificity, Western blot, protein quantification

## Abstract

G protein-coupled receptors (GPCRs) are regulated by GPCR kinases (GRKs) which phosphorylate intracellular domains of the active receptor. This results in the recruitment of arrestins, leading to desensitization and internalization of the GPCR. Aside from acting on GPCRs, GRKs regulate a variety of membrane, cytosolic, and nuclear proteins not only via phosphorylation but also by acting as scaffolding partners. GRKs’ versatility is also reflected by their diverse roles in pathological conditions such as cancer, malaria, Parkinson’s-, cardiovascular-, and metabolic disease. Reliable tools to study GRKs are the key to specify their role in complex cellular signaling networks. Thus, we examined the specificity of eight commercially available antibodies targeting the four ubiquitously expressed GRKs (GRK2, GRK3, GRK5, and GRK6) in Western blot analysis. We identified one antibody that did not recognize its antigen, as well as antibodies that showed unspecific signals or cross-reactivity. Hence, we strongly recommend testing any antibody with exogenously expressed proteins to clearly confirm identity of the obtained Western blot results. Utilizing the most-suitable antibodies, we established the Western blot-based, cost-effective simple tag-guided analysis of relative protein abundance (STARPA). This method allows comparison of protein levels obtained by immunoblotting with different antibodies. Furthermore, we applied STARPA to determine GRK protein levels in nine commonly used cell lines, revealing differential isoform expression.

## 1. Introduction

The G protein-coupled receptor kinases (GRKs) were discovered as cytosolic, membrane-associated serine/threonine kinases. Through phosphorylation of ligand-activated G protein-coupled receptors (GPCRs), GRKs enable the binding of arrestins and induce desensitization as well as internalization of the receptor [1]. The human genome encodes seven GRKs (GRK1-7) that are grouped into three subfamilies: the visual GRK subfamily (GRK1 and 7), the GRK2 subfamily (GRK2 and 3), and the GRK4 subfamily (GRK4, 5, and 6). Four of those GRKs, namely GRK2, 3, 5, and 6 are reported to be ubiquitously expressed [2].

Today, GRKs are not only known to phosphorylate GPCRs, but have also been demonstrated to act on other substrates, such as receptor tyrosine kinases, cytoplasmic kinases (e.g., src-family kinases), and even nuclear proteins [3,4,5]. Moreover, GRKs were reported to have phosphorylation-independent scaffolding properties [6,7,8]. All these activities together explain why alterations of GRK expression levels are found to be important in many pathological conditions, such as cancer, malaria, Parkinson’s-, cardiovascular-, and metabolic disease [9,10,11,12].

Taking a closer look at the distribution of the so-called ubiquitously expressed GRKs in specific tissues, it becomes apparent that there are actually striking differences in the expression levels when comparing mRNA data [13]. However, the relationship between mRNA abundance and actual expressed protein level is not always linear and depends on many different factors, such as availability of components for biosynthesis or proteasomal degradation [14,15]. In standard laboratory procedures, Western blot is a commonly used technique to investigate the actual protein level expressed in cells or tissues. Various companies offer antibodies that are advertised to specifically detect certain proteins. Unfortunately, in addition to the intended protein, many of these commercially available antibodies cause unspecific background bands, which leads to difficult interpretation; in the worst case, some antibodies fail to detect their target protein at all [16,17,18,19,20,21]. Here, we investigated the ability of eight different anti-GRK antibodies to detect the targeted GRK isoform and possible cross-reactivity against other GRK family members. We have created expression constructs for all four ubiquitously expressed human GRKs (GRK2, 3, 5, and 6) in various isoforms (Table 1), including versions with point mutations rendering them catalytically inactive (“kinase dead”: GRK2-K220R, GRK3-1-K220R, GRK5-K215R, and GRK6-1-K215R). We utilized these expression plasmids to overexpress the GRKs in HEK293 cells and determined the ability of selected commercially available antibodies to detect the proteins.

The use of validated isoform-specific antibodies to detect protein levels in cell lines or primary tissues allows the comparison of protein levels of one specific protein, but the direct comparison of expression levels of proteins or isoforms using different antibodies is not possible: antibody concentration, specificity, and clonality might have unpredictable influences on the result, e.g., a higher concentration of one antibody would result in more signal, although the protein content was identical. To overcome this problem, we established the cost-effective simple tag-guided analysis of relative protein abundance (STARPA). This method allows the relative quantification of different protein isoforms via Western blotting with validated antibodies.

## 2. Results

Lysates of HEK293 cells with overexpression of GRK2, GRK3-1, GRK3-2, GRK5, GRK6-1, GRK6-2, GRK6-3, and GRK6-4 were prepared and analyzed using eight different commercially available GRK antibodies as listed in Table 1.

**Figure 1:**
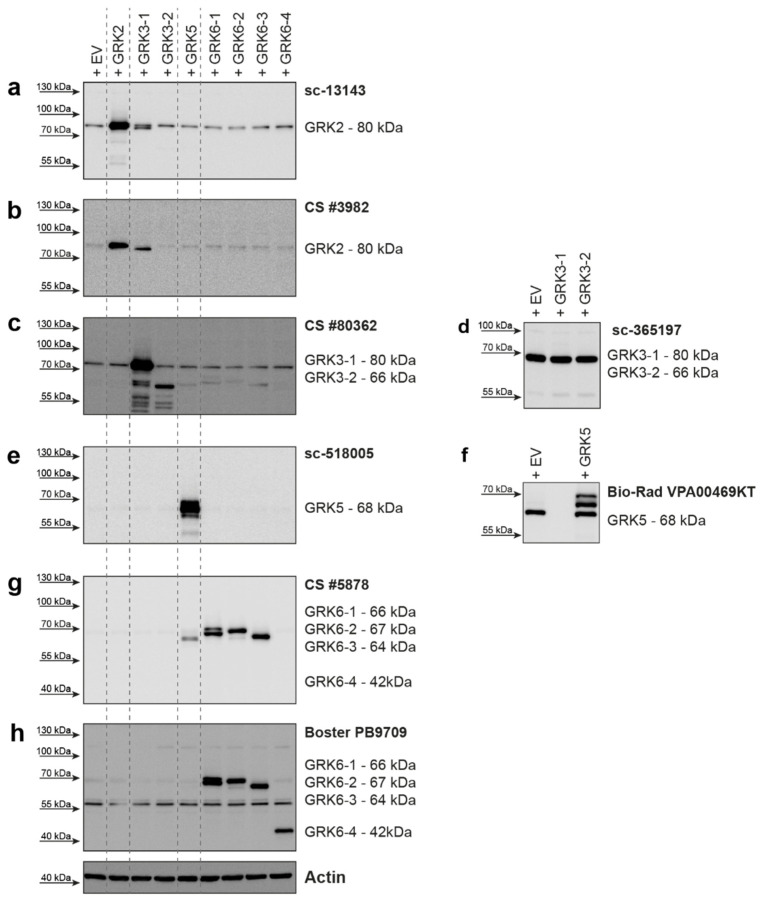
GRK isoform specificity of several commercially available GRK antibodies. HEK293 cells were transiently transfected with pcDNA3 plasmids encoding either GRK2, GRK3-1, GRK3-2, GRK5, GRK6-1, GRK6-2, GRK6-3, or GRK6-4 as indicated. GRK isoform specificity of indicated GRK antibodies was investigated in Western blot analysis. Representative blots are shown. Two different antibodies raised against GRK2 (**a**,**b**), GRK3 (**c**,**d**), GRK5 (**e**,**f**), and GRK6 (**g**,**h**) were used for the analysis. Approximate molecular weight of each antigen is stated. Detailed information on each antibody examined is listed in Table 1. Actin served as loading control.

For GRK2, two different antibodies were tested (Figure 1a,b). The Santa Cruz Biotechnology antibody raised against GRK2 (Figure 1a) detected overexpressed GRK2 and endogenous levels of GRK2, with some detection of GRK3-1. The Cell Signaling Technology antibody #3982 (Figure 1b) was also able to detect overexpressed GRK2, but the signal for endogenously expressed GRK2 was weak. This antibody detected overexpressed GRK3-1 with a stronger signal than the Santa Cruz antibody. The differences in the cross-reactivity of these two different GRK2 antibodies with the GRK3-1 protein are very surprising, as the alignment of the GRK2 epitope regions compared to the GRK3-1 sequence showed 78% (Santa Cruz antibody, Supplementary Figure S1a) or 77% (Cell Signaling Technology antibody, Supplementary Figure S1b) identical amino acids. While the similarity between these two GRK isoforms can explain the occurrence of cross-reactivity, the relative difference in the extent of cross-reactivity cannot be explained by this alignment.

The overexpression of GRK3-1 and GRK3-2 was detected by the Cell Signaling Technology antibody #80362 (Figure 1c). This antibody also mildly detected overexpressed GRK5, GRK6-1, 6-2, and 6-3 isoforms, although only 34–35% of the amino acids in the epitope region match (Supplementary Figure S1c). The Santa Cruz Biotechnology antibody sc-365197 was not able to detect the overexpressed GRK3 isoforms, but it gave a strong background band in all HEK293 lysates slightly below the 70 kDa marker (Figure 1d).

Overexpressed GRK5 protein was strongly detected by Santa Cruz Biotechnology sc-518005 antibody with no visible cross-reactivity to overexpressed GRK6 isoforms (Figure 1e). A second tested antibody from Bio-Rad (VPA00469KT) was also able to detect overexpressed GRK5, but additionally displayed a strong background band slightly below the specific protein band (Figure 1f). Our findings were reported to the supplier and the antibody was discontinued.

We created expression plasmids for four different GRK6 isoforms. GRK6-1, GRK6-2, and GRK6-3 only differ in the 30 amino acids on the C-terminus, whereas GRK6-4 is a 210 amino acid N-terminally truncated version of GRK6-1. We tested two antibodies for this GRK. The antibody from Cell Signaling Technologies (#5878) raised against the N-terminus of GRK6-1, which is identical in GRK6-2 and 6-3, was able to detect overexpressed proteins of these isoforms, but was unable to detect GRK6-4 (Figure 1g), as this isoform does not include the epitope of this antibody. We observed a slight cross-reactivity with overexpressed GRK5, which has 59% similarity in the amino acid sequence surrounding glutamate 89 of GRK6, which, according to the supplier, was used as the antigen for antibody generation (Supplementary Figure S1d). The second antibody we tested from Boster Biological Technology (PB9709) was raised against amino acids 382-417 of GRK6. This region is also present in GRK6-4. As expected, this antibody also detected GRK6-4 and the isoforms GRK6-1, 6-2, and 6-3. However, it gave a strong unspecific band around 55 kDa (Figure 1h).

The Western blots of overexpressed GRK5 and GRK6-1 (Figure 1e–h) revealed double bands of the proteins. Notably, GRKs are known to be (auto)phosphorylated [4,22,23]. To clarify whether the identified size shift was caused by phosphorylation of the GRKs, we transfected HEK293 cells with either GRK2, 3, 5, and 6 isoforms or with the catalytically inactive variants GRK2-K220R, GRK3-1-K220R, GRK5-K215R, and GRK6-1-K215R. These cells were then lysed in the presence of phosphatase inhibitors (+) or in the absence of EDTA and phosphatase inhibitors (−) to allow endogenous phosphatases to dephosphorylate the proteins while preparing the lysates. In samples overexpressing GRK2 (Supplementary Figure S2a) or GRK3 (Supplementary Figure S2b), we could not detect an influence of the endogenous phosphatases or the expression of the kinase-dead mutants on the Western blot pattern. Since the upper band is strongly diminished in the lysates with the active phosphatases (Supplementary Figure S2c), the Western blot analysis of lysates from GRK5-overexpressing cells revealed that the upper band visible in the lysates of wild-type GRK5 treated with phosphatase inhibitors is due to phosphorylation of the kinase. The kinase-dead mutant did not show any change with or without active phosphatases. Similar findings were obtained in the case of GRK6-1 with both tested antibodies (Supplementary Figure S2d,e). These findings indicate a strong phosphorylation, which led to a size shift of GRK5 and 6 in our experimental setup.

**Figure 2:**
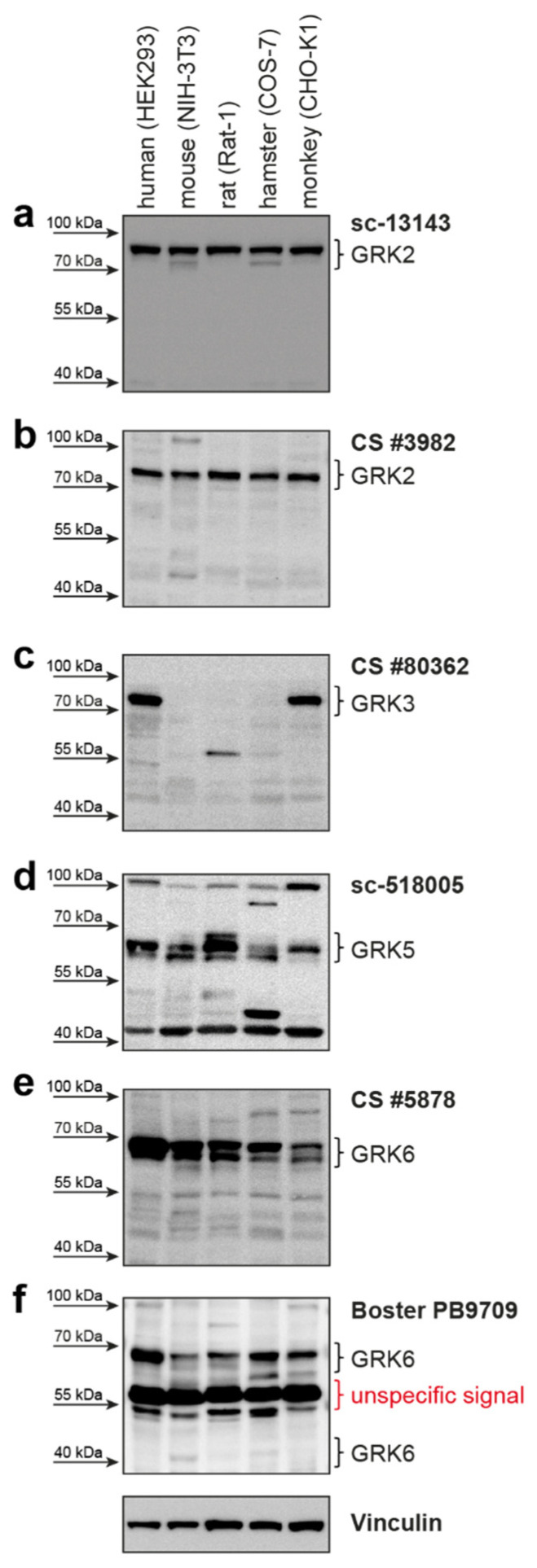
Species reactivity of tested antibodies. (**a**–**f**) Western blot analysis of HEK293 (human), NIH-3T3 (mouse), COS-7 (hamster), Rat-1 (rat), and CHO-K1 (monkey) cell lysates using the denoted anti-GRK antibodies. Vinculin served as loading control.

The manufacturer claims the antibodies can detect the respective GRK not only in humans, but also in other species, such as mice, hamsters, rats, and monkeys (Table 1). Therefore, we tested the ability of the GRK antibodies to detect endogenously expressed GRKs in HEK293 (human), NIH-3T3 (mouse), COS-7 (hamster), Rat-1 (rat), and CHO-K1 (monkey) cell lines. All these species express different GRK isoforms homologous to the human GRKs (Supplementary Table S1). Usage of both GRK2 antibodies resulted in a clear signal corresponding to the size of human GRK2 in all analyzed cell lines (Figure 2a,b). The amino acid sequence alignment in the respective regions of antibody recognition shows 96% to 100% identity to the human GRK2 sequence (Supplementary Figure S3a,b).

The GRK3 antibody only detected the endogenously expressed GRK3 in the human and monkey cell lines (Figure 2c). These two species have the identical amino acid sequence in the antibody detection region. The amino acid sequences of mouse, rat, and hamster GRK3, however, are more variable, with only 89%, 88%, and 90% identity, respectively (Supplementary Figure S4). These differences might explain the absence of a specific GRK3 band. However, GRK3 might be absent in these analyzed cell lines under our experimental conditions and is therefore not detectable.

Utilization of the GRK5 antibody leads to detectable signals in all tested cell lines from the different species (Figure 2d) at the expected protein size. The similarity to human GRK5 is 87–95% (Supplementary Figure S5).

A signal corresponding to the size of human GRK6 can be detected in all analyzed cell lines with both tested GRK6 antibodies (Figure 2e,f). Notably, there is no clearly visible band reflecting the GRK6-4 isoform (Figure 2f). The GRK6 sequences of the different species are very similar to human GRK6-1—ranging from 92% to 96% similarity for the epitope region of the Cell Signaling Technology antibody (Supplementary Figure S6a) and 88–100% similarity for the Boster Biological Technology antibody (Supplementary Figure S6b).

**Figure 3:**
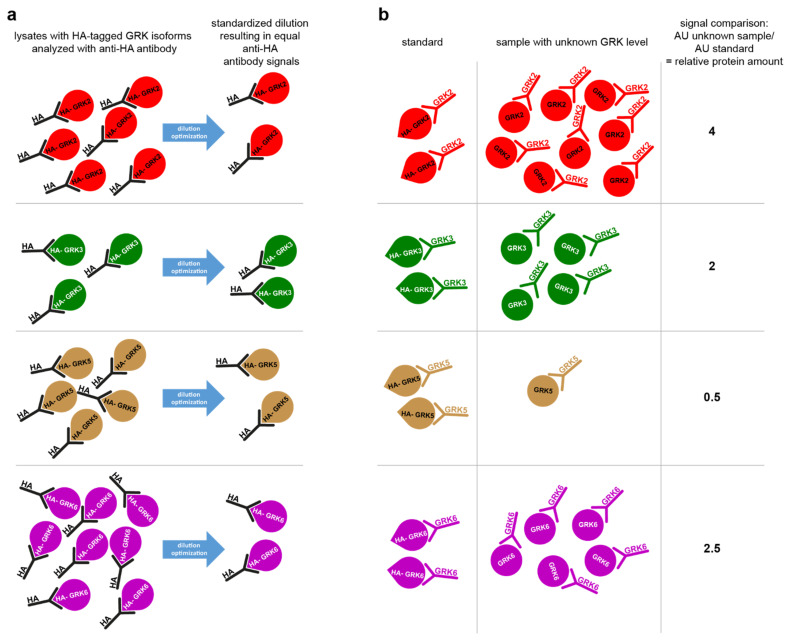
Simple tag-guided analysis of relative protein abundance (STARPA). Schematic depiction of STARPA implementation (**a**) and functionality (**b**). The creation of a protein standard for each GRK isoform containing equal amounts of HA-tagged GRK (**a**) enables the analysis of relative GRK levels via Western blot in any sample containing untagged GRK using GRK isoform-specific antibodies (**b**). AU, arbitrary units.

Next, using our quadruple GRK knockout HEK293 cells (ΔQ-GRK, lacking endogenous expression of GRK2, 3, 5, and 6 [24]), we established the cost-effective simple tag-guided analysis of relative protein abundance (STARPA). This method allows the relative quantification of GRK protein amount in Western blot samples with unknown GRK isoform expression levels. Figure 3a,b schematically depicts the principle STARPA concept: after transfection of the hemagglutinin-tagged GRK (HA-GRK) isoforms, cell lysates are analyzed for their anti-HA-antibody signal, and the optimal dilution factor to result in equal signals for all HA-GRK isoforms is experimentally determined by sample dilution (Figure 3a). These standardized samples are loaded onto the gel as references alongside the lysates with unknown GRK expression. Utilizing the GRK-specific antibodies, the normalized signal will reflect the relative amount of this specific isoform (Figure 3b), Equation (1):

(1) $$\frac{d_{A}\left(U\right)}{d_{A}\left(S_{a}\right)}=y_{a}$$

where $y_{a}$ is the relative amount of protein a in the unknown sample U, and $d_{A}$ is the densitometric signal acquired with antibody A in the STARPA standard of protein a ($S_{a})$, all measured on the same blot. For example:

$$\frac{anti\text{-}GRK3\;signal\;of\;unknown\;sample}{anti\text{-}GRK3\;signal\;of\;HA\text{-}GRK3\;standard}=relative\;GRK3\;expression$$

**Figure 4:**
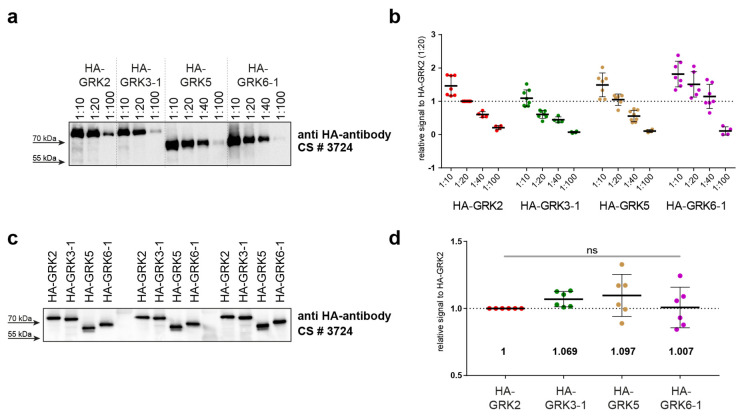
Implementation of protein standards for STARPA. (**a**) To create the HA-tagged GRK protein standards for STARPA, ΔQ-GRK cells (HEK293 cells with knockout of GRK2, 3, 5, and 6, described in [24]), were transiently transfected with HA-tagged GRK2, 3-1, 5, or 6-1. Serial dilutions of lysates were analyzed for their GRK levels using anti-HA antibody. A representative blot is shown. (**b**) Blots were quantified, and the relative signal compared to the respective signal in the 1:20 dilution of HA-GRK2 are shown as dot plots with corresponding means ± standard deviation (SD), indicated by bars. (**c**) According to the results shown in (**b**), new dilutions from the same lysates were created with the aim to contain equal protein concentrations of the corresponding HA-GRK isoform. Their HA-GRK level was determined via Western blot analysis using anti-HA antibody. A representative blot showing 3 of 6 datasets is shown. (**d**) Blots were quantified, and the relative signals compared to the respective signals in the HA-GRK2 of each dataset are shown as dot plots with corresponding means ± SD, indicated by bars. Additionally, means are stated below the dot plots. No statistical differences (ns) were found using one-way ANOVA and Tukey’s test.

First, we prepared lysates of overexpressed HA-tagged GRK isoforms in ΔQ-GRK cells, as these are devoid of endogenous GRK expression. Therefore, any Western blot signal of GRKs would be caused by the overexpressed HA-GRK versions, and no purification steps were necessary. We analyzed a dilution series ranging from 1:10 to 1:100 by Western blotting using an anti-HA antibody (Figure 4a). Quantification of multiple analyses of several dilutions allowed the estimation of optimal standard dilutions (Figure 4b). In our setup, we chose the HA-GRK2 1:20 dilution as reference. In order to minimize differences in developing times between different blots, all signals obtained for the individual blots were calculated relative to this HA-GRK2 signal. We subsequently identified the dilutions of the other GRK isoforms that displayed the smallest differences in HA signals compared to our GRK2 reference dilution (namely GRK3 1:10, GRK5 1:20, and GRK6 1:40). Next, new dilution series for each GRK were prepared in order to more finely optimize results; these were again analyzed by immunoblotting (data not shown). The best-matching dilutions were then extensively validated (Figure 4c,d).

**Figure 5:**
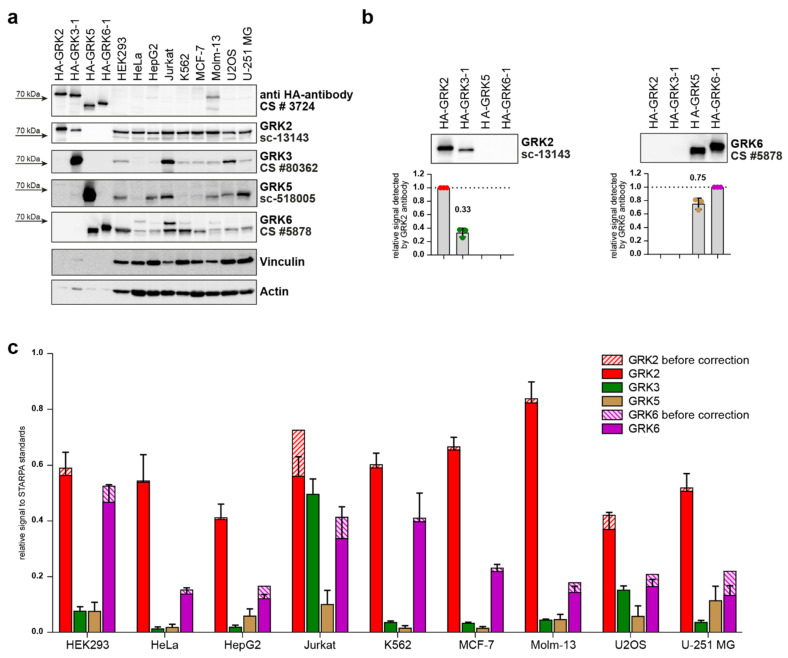
STARPA reveals differential GRK expression in commonly used cell lines. (**a**) STARPA was used to determine relative GRK expression in HEK293, HeLa, HepG2, Jurkat, K562, MCF-7, Molm-13, U2OS, and U-251 MG cells. Lysates of different cell lines together with the validated STARPA standards (Figure 4c,d) were loaded onto polyacrylamide gels. Western blot analysis using anti-HA antibody confirmed the STARPA standards. Immunoblotting using anti-GRK antibodies for each isoform allowed the determination of endogenously expressed GRK levels in the cell lines. Shown are representative images of three independent blots from identical lysates. (**b**) Close ups of the STARPA standards from the immunoblot presented in (**a**) displaying the cross-reactivity of the anti-GRK2 and anti-GRK6 antibody towards GRK3 and GRK5, respectively. Below, the quantification of three independent blots ± SD. The means of the obtained signals are indicated and were used to determine the respective cross-reactivity coefficient of the antibodies. (**c**) STARPA blots were quantified according to each GRK isoform-specific antibody, and the relative signal compared to the respective signal in the standards is shown as mean + SD of three independent blots from identical lysates. The calculated relative expression levels of GRK2 and GRK6 before (striped) and after (solid) cross-reactivity correction are presented.

These validated standards were then used to determine relative GRK protein levels in nine different commonly used cell lines. Thus, standards were loaded onto Western blot gels in parallel with 30 µg of total protein from HEK293, HeLa, HepG2, Jurkat, K562, MCF-7, Molm-13, U2OS, and U-251 MG cell lysates with unknown GRK content (Figure 5a). The samples were analyzed with GRK-specific antibodies as indicated, and additionally with anti-HA antibody. The previously described cross-reactivity of the utilized GRK2 (sc-13143) and GRK6 (CS #5878) antibodies against GRK3 and GRK5, respectively, while weakly observed in the non-standardized lysates (Figure 1a,g; Table 1), were clearly visible in the standards with an equal amount of HA-GRK (Figure 5a,b). Quantification of GRK3 signal detected by the GRK2 antibody revealed cross-reactivity resulting in a 33% signal, and for GRK5 detection by GRK6 antibody a 75% signal (Figure 5b). These levels of cross-reactivity need to be considered when calculating relative protein levels using STARPA. Therefore, the cross-reactivity was quantified as a cross-reactivity coefficient for antibody $B\;(c_{B})$ using the STARPA standards, Equation (2):

(2) $$\frac{d_{B}\left(S_{a}\right)}{d_{B}\left(S_{b}\right)}=c_{B}$$

where the densitometric signal of STARPA standard a detected by antibody B ($d_{B}\left(S_{a}\right)$) divided by the densitometric signal of STARPA standard b detected by antibody B ($d_{B}\left(S_{b}\right)$) results in the cross-reactivity coefficient for antibody B towards protein a ($c_{B}$). For example, calculating the cross-reactivity coefficient for the anti-GRK2 antibody (sc-13143) exhibiting cross-reactivity towards GRK3 ($c_{GRK2}$):

$$\frac{anti\text{-}GRK2\;signal\;of\;HA\text{-}GRK3\;standard}{anti\text{-}GRK2\;signal\;of\;HA\text{-}GRK2\;standard}=c_{GRK2}$$

Consequently, when determining the relative protein content of a specific protein using antibody B, which cross-reacts with protein a, the real protein content of b can only be calculated if the amount of a is subtracted accordingly. Thus, Equation (1) is modified to include the cross-reactivity coefficient, Equation (3):

(3) $$\frac{d_{B}\left(U\right)}{d_{B}\left(S_{b}\right)}-y_{a}*c_{B}=y_{b}$$

where $y_{b}$ is the relative amount of protein b in sample U corrected for the cross-reactivity of antibody B towards protein a. Continuing the example above:

$$\frac{anti\text{-}GRK2\;signal\;of\;unknown\;sample\;}{anti\text{-}GRK2\;signal\;of\;HA\text{-}GRK2\;standard}-relative\;GRK3\;expression\;*\;c_{GRK2}\;\;\;\;\;\;\;\;\;\;\;\;\;\;\;\;\;\;=corrected\;relative\;GRK2\;expression$$

After quantification of the immunoblots, the cell line signals obtained from incubation with GRK-specific antibodies were divided by the signal with the GRK-specific antibody of the respective standard (Equation (1)), resulting in the relative GRK expression pattern before correction of the analyzed cell lines (Figure 5c). Subsequently, the obtained relative expression of GRK2 and GRK6 were corrected for cross-reactivity signals as described above (Equations (2) and (3), Figure 5c). Notably, the impact of this correction is reduced if GRK3 or GRK5 protein levels are low.

GRK2 was found to be the most expressed isoform in all nine tested cell lines. Although GRK6 was the second most abundant isoform in all cell lines except for Jurkat cells, its expression compared to the respective GRK2 level varied between the cell lines. While GRK6 protein levels reach up to 83% of GRK2 in HEK293 cells, our analysis showed that GRK6 expression is much lower in HeLa, HepG2, MCF-7, Molm-13, and U2OS cells, reaching between 18% (Molm-13) and 34% (MCF-7) of the respective GRK2 level.

In five out of nine cell lines, the measured GRK3 and GRK5 protein levels were less than a third of the respective GRK2 or GRK6 expression. In HepG2, Jurkat, U2OS, and U-251 MG cells, however, GRK3 and GRK5 levels differed. GRK5 reached 49% and 86% of the recorded GRK6 expression in HepG2 and U-251 MG cells, respectively. U2OS cells expressed similar protein levels of GRK3 and GRK6 (92% of GRK6 expression). In Jurkat cells, GRK3 was the second most abundant isoform, reaching 89% of the GRK2 protein level, in contrast to the remaining analyzed cell lines.

Taken together, the employment of STARPA demonstrates differential GRK isoform expression in these commonly utilized cell lines.

## 3. Discussion

Most of the tested antibodies (GRK2: sc-13143 and CS #3982; GRK3: CS #80362; GRK5: sc-518005 and VPA00469KT; GRK6: CS #5878 and PB9709) are able to detect the targeted protein, but some also strongly label background bands with similar protein size, leading to difficult interpretation of the expression levels, especially at endogenous levels (GRK5: VPA00469KT; GRK6: PB9709). One antibody (sc-365197, GRK3) did not detect its target GRK at all; it detected only a very strong background band (Figure 1d). In our test setup, we also observed cross-reactivity with the other overexpressed GRK isoforms. This cross-reactivity is also the reason why the four standards used for STARPA could not be mixed into one standard containing all GRK isoforms. We could detect GRK3 while using GRK2 antibodies and GRK5 when using GRK6 antibody (Figure 1a,b,g; Figure 5a,b). This observed cross-reactivity should be taken into consideration, as the influence on the obtained result depends on the expression ratio of GRK3 to GRK2 and GRK5 to GRK6.

Apart from detecting more than one GRK isoform, a given antibody could possibly detect the same GRK in other species, as GRKs are conserved among mammals [25]. To test whether the antibodies would recognize the proteins of mice, rats, hamsters, or monkeys, we analyzed cell lines of these species. For GRK2, GRK5, and GRK6, we detected bands in the size comparable to the human GRKs, but in the case of GRK3, we did not observe a signal in mouse, rat, or hamster cells, although the supplier states the recognition of mouse GRK3. The absence of detectable protein in the tested cell lines could be either due to the lack of antibody affinity to the species-typical GRK, or simply due to the absence of that GRK in the tested cell line. To clearly validate the recognition of GRKs from different species, it would be mandatory to test the antibodies with overexpressed isoforms of the species of interest, and/or in protein lysates of knockout (KO) animals or cell lines.

**Figure 6:**
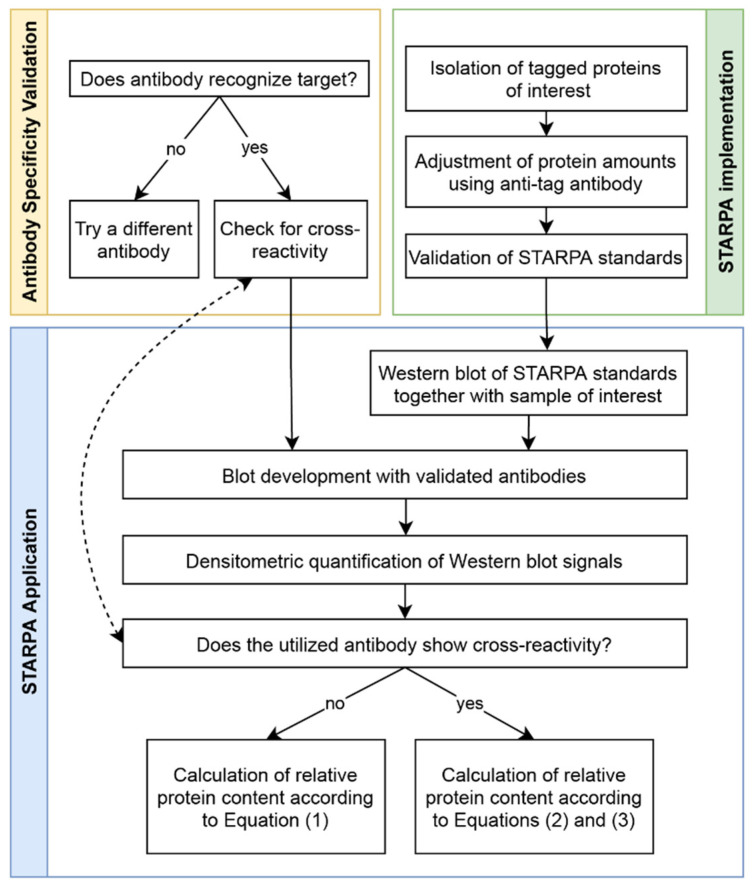
Sequence of events needed to conduct STARPA experiment. Antibody validation (orange) and STARPA implementation (green) are prerequisites to applying STARPA (blue).

Utilizing the validated anti-GRK antibodies, we established STARPA as an economical method for relative quantification of GRK isoform levels. The sequence of events for STARPA are summarized in Figure 6. Due to the cross-reactivity of GRK2 and GRK6 antibodies as mentioned above, recorded levels of these isoforms must be corrected. In order to calculate the real amount of GRK2 or 6, the quantification of GRK3 or 5 respectively needs to be carried out as well, using an antibody without detectable cross-reactivity against the other isoforms. For protein samples where GRK2 or GRK6 levels are much higher than GRK3 or GRK5 levels, respectively, the correction could be neglected (Figure 5c). The protein standards are perfectly suited to determine the cross-reactivity of the utilized antibody, since the level of cross-reactivity could be over- or underestimated depending on the expression level of the different proteins under endogenous conditions.

Our analysis of nine commonly used cell lines demonstrates the ability of STARPA to reveal the differential protein expression of the GRK isoforms in cells derived from various tissues. Notably, the here-reported differences and expression patterns reflect a snapshot of the GRK levels in our growth conditions. GRK levels can change during progression of the cell cycle [26], or the expression pattern might also be influenced by the level of available nutrients, cell density, plasticware, cell passage, and other factors [27]. Taking all these factors into account, the expression level of a given cell line might vary between different laboratories and must be individually analyzed.

Differences in GRK expression levels in a given cellular context potentially lead to changes in downstream signaling and trafficking of GPCRs. The knockdown of GRK2/3 or GRK5/6 differentially influenced downstream extracellular-signal regulated kinases (ERK) signaling of angiotensin II type 1 receptor (AT1R) or vasopressin 2 receptor (V2R) [28,29]. Additionally, some GPCRs can only be internalized upon ligand stimulation if specific GRKs are expressed. For internalization of the muscarinic acetylcholine receptor 5 (M5R) and µ-opioid receptor (MOP) the presence of either GRK2 or 3 is mandatory [24]. GPCRs are involved in numerous diseases, and GRK expression alterations have been reported for several pathophysiological conditions. In heart failure for example, the upregulation of GRK2 and GRK5 leads to the downregulation and desensitization of the beta adrenergic receptors [30]. Hence, investigation of the impact of GRK levels on GPCR signaling becomes essential [13]. While the expression of each isoform can be compared based on the mRNA level using standardized real-time quantitative PCR, the here-described STARPA method now allows this comparison at the protein level to obtain specific GRK expression patterns.

In this study we describe STARPA as a method that can be conducted using only standard laboratory procedures and equipment to determine relative GRK protein levels, given that antibodies recognizing the protein are available and appropriately validated. As availability of antibodies targeting a protein of interest might be limited, biologicals—as many of them are clinically used antibodies [31]—or the newly developed nanobodies [32] could be additionally tested.

By introducing standards, the variability of antibody concentration and affinity can be normalized and thereby allow direct comparison. Of note, all samples and the standards need to be loaded onto one Western blot gel, and the blots need to be conducted multiple times from the identical lysate to obtain reliable results. This limits the sample number that can be processed in one experimental setup.

STARPA enables the relative comparison of different expression levels of any protein that can be expressed with the same protein tag. We anticipate that STARPA is not limited to cytosolic proteins but could be applied to any protein class. Notably, GPCRs are also differentially expressed in different tissues or cancer [33,34]. Studies of GPCR protein levels in relation to other GPCRs, GRKs, β-arrestins, G proteins, or any other protein could be conducted using STARPA.

We used the ΔQ-GRK cell line with a KO of GRK2, 3, 5, and 6 [24] for expression of HA-GRK isoforms. In that case, no purification of the HA-tagged GRKs was needed, as there is no endogenous GRK present. If no KO cells are available to overexpress the desired protein, an immunoprecipitation of the tagged proteins should be carried out before the creation of the dilution series.

Taken together, we strongly recommend testing any antibody with exogenously expressed proteins to clearly confirm identity of the obtained Western blot results. Furthermore, we propose STARPA as a cost-effective method using standard laboratory equipment to compare relative levels of different proteins.

## 4. Materials and Methods

### 4.1. Expression Constructs

Expression pcDNA3 plasmids for human GRK2 (NP_001610.2), GRK3-1 (NP_005151), GRK5 (NP_005299.1), and GRK6-1 (NP_001004106.1), as well as the kinase-dead mutants GRK2-K220R, GRK5-K215R, and GRK6-1-K215R were previously described [24]. The remaining isoforms GRK3-2 (NP_001349707), GRK6-2 (NP_002073.2), GRK6-3 (NP_001004105.1), and GRK6-4 (NP_001351093.1) were established from cDNA isolated from HEK293 cells. The K220R mutant of GRK3-1 was created by site-directed mutagenesis of the wild-type plasmid. For the N-terminally HA-tagged GRKs, the encoding sequence for the peptide MYPYDVPDYA was inserted before the start codon of the respective GRK. The identity of all plasmids was confirmed by sequencing. PubMed accession numbers for cDNA transcripts and proteins of all species are summarized in Supplementary Table S1.

### 4.2. Cell Lines

All cell lines were regularly checked for mycoplasma infection using the Lonza (Basel, Switzerland) MycoAlert mycoplasma detection kit (LT07-318) and were found to be negative.

HEK293 (human, German Collection of Microorganisms and Cell Cultures GmbH, Braunschweig, Germany (DSMZ Germany), ACC 305), NIH-3T3 (mouse, DSMZ Germany, ACC 59), COS-7 (hamster, DSMZ Germany, ACC 60), Rat-1 (rat, American Type Culture Collection, Manassas, VA, USA (ATCC), CRL-2210), and U2OS (human, kind gift from S. Hoffmann and M. Fischer, Leibniz-Institut für Alternsforschung—Fritz-Lipmann-Institut e.V. (FLI) Jena, Germany), and U-251 MG (human, kind gift from C. Mawrin, Otto-von-Guericke Universität, Magdeburg, Germany) cells were cultured in Dulbecco’s Modified Eagle’s Medium (DMEM, Sigma-Aldrich, Taufkirchen, Germany, D6429) supplemented with 10% fetal calf serum (FCS, Sigma-Aldrich F7524) and 1% penicillin streptomycin mixture (Sigma-Aldrich, Taufkirchen, Germany, P0781). CHO-K1 (monkey, DSMZ Germany, ACC 110) cells were cultured in DMEM/F-12 (Life Technologies, Bleiswijk, The Netherlands, 21041-025) supplemented with 10% FCS and 1% penicillin streptomycin mixture. The cell lines HeLa (human, DSMZ Germany, ACC 57), K562 (human, DSMZ Germany ACC 10), Jurkat (human, DSMZ Germany ACC 282), and Molm-13 (human, DSMZ Germany ACC 554) were cultured in RPMI1640 (Sigma-Aldrich, Taufkirchen, Germany, R8758) supplemented with 10% heat-inactivated FCS and 1% penicillin streptomycin mixture. HepG2 (human, DSMZ Germany ACC 180) cells were cultured in DMEM (Sigma-Aldrich, Taufkirchen, Germany, D6429) supplemented with 10% heat-inactivated FCS, and 1% penicillin streptomycin mixture. MCF-7 (human, kind gift from H. Pospiech, Leibniz-Institut für Alternsforschung—Fritz-Lipmann-Institut e.V. (FLI) Jena, Germany) cells were cultured in DMEM containing 4.5 g/L Glucose and 3.7 g/L sodium carbonate (PAN Biotech, Aidenbach, Germany, P04-03500) supplemented with 10% FCS, 1 mM sodium pyruvate and 2 mM L-glutamine. Our HEK293 derivative with knockout of GRK2, 3, 5, and 6 (ΔQ-GRK) is described in [24].

### 4.3. Antibody Validation

For the antibody specificity testing, 7 × 10^5^ HEK293 cells were seeded in each well of a 6-well plate and were transfected with 2 µg of respective plasmid DNA using polyethylenimine (PEI) reagent (Sigma-Aldrich, Taufkirchen, Germany, 408727). After 24 h, cells were washed once with cold phosphate buffered saline (PBS, Sigma-Aldrich, Taufkirchen, Germany, P4417) and lysed with RIPA lysis buffer (1% NP-40, 1 mM Ethylenediaminetetraacetic acid (EDTA), 50 mM Tris-HCl pH 7.4, 150 mM sodium chloride (NaCl), and 0.25% sodium-deoxycholate) including protease (Roche 04693132001) and phosphatase inhibitors (Roche 04906845001) diluted in RIPA buffer according to the manufacturer’s recommendations. For the experiments without phosphatase inhibitors, the RIPA buffer was prepared without EDTA and only supplemented with protease inhibitors diluted in this EDTA-free buffer. To the cleared lysates, 20 µL of 6× sample buffer (375 mM Tris-HCl pH 6.8, 12% Sodium Dodecyl Sulfate (SDS), 30% glycerol, 500 mM Dithiothreitol (DTT)) were added per 100 µL lysate. Samples were boiled for 5 min at 95 °C and equal amounts of the lysates were loaded onto polyacrylamide gels and blotted onto nitrocellulose membranes. The membranes were blocked with 5% dry milk in 1× Tris buffered saline with Tween 20 (TBST) (10× TBST:200 mM Tris-HCl pH 7.6, 1.37 M NaCl, and 10 mL Tween 20), and incubated in the diluted primary antibodies (all diluted in 5% bovine serum albumin in 1× TBST) overnight at 4 °C with gentle shaking. The utilized GRK antibodies are listed in Table 1, the Boster Biological Technology GRK6 antibody PB9709 was obtained from Antibodies-Online GmbH, Aachen, Germany with ordering number ABIN3042435, actin antibody was purchased from Sigma-Aldrich (Taufkirchen, Germany, A5441, dilution 1:5000), vinculin antibody was obtained from Biozol (Eching, Germany, BZL03106, dilution 1:1000). After washing with 1× TBST, the membranes were incubated in 5% dry milk in 1× TBST of the respective secondary horseradish peroxidase (HRP)-coupled antibody (sera care, Milford, MA, USA, anti-rabbit #5220-0336, and anti-mouse #5220-0341; both 1:10,000) and analyzed using a Fujifilm Life Science USA LAS4000.

### 4.4. STARPA

For the quantitative analysis, 5 × 10^6^ ΔQ-GRK HEK293 cells were seeded per 10 cm dish and transfected with 20 µg of the respective HA-tagged GRK expression plasmid using PEI reagent. Cells were lysed with 1 mL of RIPA buffer (containing phosphatase and protease inhibitors) and processed as described above. The obtained lysates were supplemented with sample buffer as described above, and further diluted with 1× sample buffer as indicated. Multiple Western blots were performed as described above using an anti-HA antibody (Cell Signaling Technology, Frankfurt am Main, Germany, #3724, dilution 1:1000). Quantification was performed using the Fujifilm Multi Gauge software V3.0.

For analysis of the endogenous GRK expression, cells were cultivated in full medium, washed with cold PBS, and lysed in RIPA containing protease and phosphatase inhibitors. The total protein concentration of the cleared lysates was determined using a BCA Protein-Assay (Thermo Scientific, Bleiswijk, The Netherlands, 23225), and 30 µg of protein was loaded per lane and further processed as described above.

Statistical analysis was conducted in R 4.0.3. A type I error probability of 0.05 was considered to be significant in all cases.

## Figures and Tables

**Table 1:** List of eight commercially available antibodies examined in this study, targeting the ubiquitously expressed human GRK isoforms. Overview of the tested antibodies (monoclonal (mc), polyclonal (pc)) and the tested GRK isoform is provided, including the supplier’s information, our review of each antibody, and the dilution used in Western blot to determine the antibody specificity. Additionally, the number of amino acids (aa) and the calculated approximate molecular weight (MW) for each human GRK isoform are listed. Accession numbers for protein isoforms can be found in the Materials and Methods section and additionally summarized in Supplementary Table S1.

	No. of aa and MW	Supplier’s Information				Our Findings	
		Ordering no. and Species	Supplier	Antigen	Species Reactivity	Dilution	Performance
**GRK2**	689 aa 80 kDa	sc-13143 (C-9) mouse mc	Santa Cruz Biotechnology	raised against amino acids 468-689 of human GRK2	human, mouse, rat	1:500	good detection of endogenous GRK2 in HEK293; cross-reactivity with GRK3
**GRK2**	689 aa 80 kDa	CS #3982 rabbit pc	Cell Signaling Technology	immunizing animals with a synthetic peptide corresponding to the amino-terminal residues of human GRK2	human, mouse, rat, hamster, monkey	1:1000	cross-reactivity with GRK3; only weak detection of endogenous GRK2 in HEK293 cells
**GRK3-1**	688 aa 80 kDa	sc-365197 (C-11) mouse mc	Santa Cruz Biotechnology	raised against amino acids 646-688 of human GRK3	human, mouse, rat	1:250	does not detect overexpressed human GRK3 isoforms—only background band
**GRK3-2**	575 aa 66 kDa						
**GRK3-1**	688 aa 80 kDa	CS #80362 (D8G6V) rabbit mc	Cell Signaling Technology	immunizing animals with a synthetic peptide corresponding to residues surrounding Lys454 of human GRK3	human, mouse	1:250	slight cross-reactivity with overexpressed GRK6 isoforms
**GRK3-2**	575 aa 66 kDa						
**GRK5**	590 aa 68 kDa	sc-518005 (D-9) mouse mc	Santa Cruz Biotechnology	raised against amino acids 94-157 of human GRK5	human, mouse, rat	1:250	good detection of endogenous GRK5 in HEK293
**GRK5**	590 aa 68 kDa	VPA00469KT rabbit pc	Bio-Rad	synthetic peptide directed towards the middle region of human GRK5	human, mouse	1:1000	strong background band; reported to supplier, was then discontinued
**GRK6-1**	576 aa 66 kDa	CS #5878 (D1A4) rabbit mc	Cell Signaling Technology	synthetic peptide corresponding to residues surrounding Glu89 of human GRK6	human, mouse, rat	1:1000	cross-reactivity with GRK5
**GRK6-2**	589 aa 67 kDa						
**GRK6-3**	560 aa 64 kDa						
**GRK6-1**	576 aa 66 kDa	PB9709 rabbit pc	Boster Biological Technology	C-terminus of human GRK6 (amino acids 382-417)	human, rat	1:1000	strong background around 55kDa; can detect human isoform 6-4
**GRK6-2**	589 aa 67 kDa						
**GRK6-3**	560 aa 64 kDa						
**GRK6-4**	366 aa 42 kDa						

## Supplementary Materials

The following are available online at: www.mdpi.com/article/10.3390/ijms23031195/s1.
