# Supplementary material for: Suitability of GRK Antibodies for Individual Detection and Quantification of GRK Isoforms in Western Blots
Source: Int J Mol Sci. 2022 Jan 21;23(3):1195. doi: 10.3390/ijms23031195 (PMC8835249; doi:10.3390/ijms23031195)
Supplement: Supplementary file 1 [file ijms-23-01195-s001.zip › ijms-1549862-supplementary.pdf]

Supplementary Figure S1

a

Antibody Santa Cruz Biotechnology (sc-13143) raised against amino acids 468-689 of human GRK2 (78 % matches)

```
huGRK2         468 pplipprgevnaadafdigsfdeedtkgiklldsdqelyrnfpltiserwqqevaetvfdtinaetdrlearkkaknkqlgheedyalgkdcimhgymskmgnpfltqwqrryfylfpnr
huGRK3-1       468 pplipprgevnaadafdigsfdeedtkgiklldcdqelyknfplviserwqqevtetvyeavnadtdkiearkraknkqlgheedyalgkdcimhgymlklgnpfltqwqrryfylfpnr

huGRK2         588 lewrgegeapqslltmeeiqsveetqikerkclllkirggkqfilqcdsdpelvqwkkelrdayreaqqlvqrvpkmknkprspvvelskvplvqrgsangl
huGRK3-1       588 lewrgegesrqnlltmeqilsveetqikdkkcilfrikggkqfvlqcesdpefvqwkkelnetfkeaqrllrrapkflnkprsgtvelpkpslchrns-ngl
```

b

Antibody Cell Signaling Technology (#3982) raised against amino-terminal residues of human GRK2
is cross-reactive with overexpressed GRK3-1 (77 % matches)

```
huGRK2           1 madleavladvsylmamekskatpaaraskkillpepsirsvmqkyledrgevtfekifsqklgyllfrdfclnhleearplvefyeeikkyekleteeervarsreifdsyimkellac
huGRK3-1         1 madleavladvsylmamekskatpaaraskrivlpepsirsvmqkylaerneitfdkifnqkigfllfkdfclneineavpqvkfyeeikeyekldneedrlcrsrqiydayimkellsc
```

c

Antibody Cell Signaling Technology (#80362) raised against amino acids surrounding lysine 454 (red) of GRK3
slightly detects overexpressed GRK6-1, 6-2, and 6-3 (35 % matches)

```
huGRK3-1       400 heidrmtltvnvelpdtfspelkslleglllqrdvskrlgchgggsqevkehsffkgvdwqhvylqkypplipprgevnaadafdigsfdeedtkgikl
huGRK6-1       395 eeverlvkevpeeyserfspqarslcsqllckdpaerlgcrggsarevkehplfkklnfkrlgagmleppfkpdpqaiyckdvldieqf--stvkgvel
```

Antibody Cell Signaling Technology (#80362) raised against amino acids surrounding lysine 454 (red) of GRK3
slightly detects overexpressed GRK5 (34 % matches)

```
huGRK3-1       400 heidrmtltvnvelpdtfspelkslleglllqrdvskrlgchgggsqevkehsffkgvdwqhvylqkypplipprgevnaadafdigsfdeedtkgikl
huGRK5         395 eevdrrvleteevyshkfseeaksickmlltkdakqrlgcqeegaaevkrhpffrnmnfkrleagmldppfvpdpravyckdvldieqfs--tvkgvnl
```

d

Antibody Cell Signaling Technology (#5878) raised against amino acids surrounding glutamic acid 69 (red) of GRK6
slightly detects overexpressed GRK5 (59 % matches)

```
huGRK6-1        30 wrqmlqfphisqceelrlslerdyhslcerqpigrllfrefcatrpelsrcvafldgvaeyevtpddkrkacgrqltqnfl
huGRK5          30 wkeilkfphisqcedlrrtidrdycslcdkqpigrllfrqfcetrpglecyiqfldsvaeyevtpdeklgekgkeimtkyl
```

Supplementary Figure S1: Alignment of protein sequences used to create designated antibodies and the corresponding sequence of the indicated GRK isoform which are also detected by the antibody. Amino acids identical in both sequences are highlighted in green. Total identity is denoted in per cent. **a** Alignment of human GRK2 and human GRK3-1 protein sequences from amino acid 468 to 689 which are both recognized by antibody sc-13143 (Santa Cruz Biotechnology). **b** Alignment of human GRK2 and human GRK3-1 protein sequences from the first amino acid to amino acid 120 which are both recognized by antibody CS #3982 (Cell Signaling Technology). **c** Alignment of human GRK3-1 with human GRK6-1 and GRK5 protein sequences from amino acid 400 to 499 and 395 to 494, respectively. Antibody CS #80362 (Cell Signaling Technology) was raised against GRK3 but also detects GRK6-1, -2, -3 and GRK5. **d** Alignment of human GRK6-1 and human GRK5 protein sequences from amino acid 30 to 499 which are both recognized by antibody CS #5878 (Cell Signaling Technology).

Supplementary Figure S2

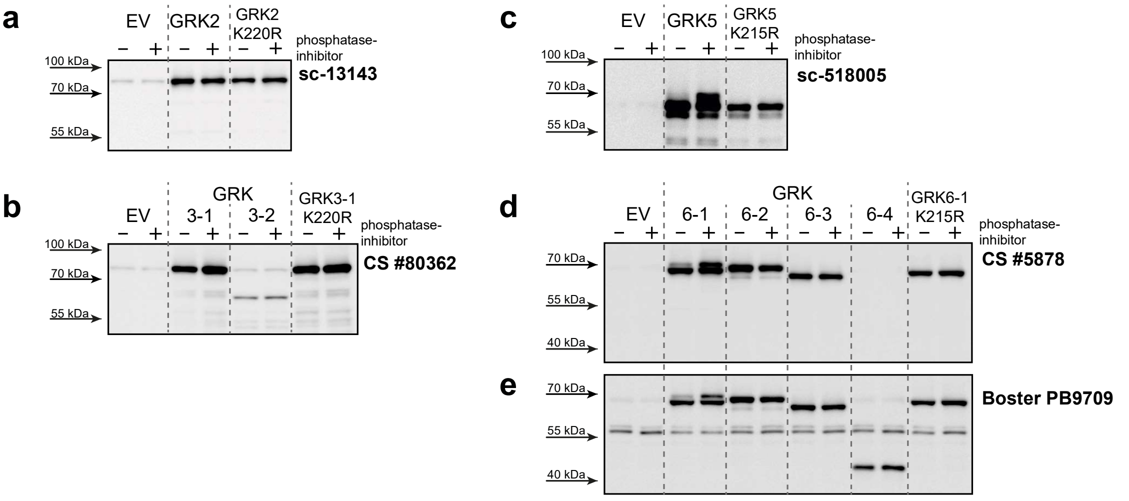

Supplementary Figure S2:
HEK293 cells were transfected with either empty vector (EV), GRK2 (**a**), 3 (**b**), 5 (**c**), and 6 isoforms (**d**) or their corresponding kinase-dead variants GRK2-K220R (**a**), GRK3-1-K220R (**b**), GRK5-K215R (**c**), and GRK6-1-K215R (**d**). Lysates were prepared in presence (+) or absence (-) of phosphatase inhibitors and EDTA. Western blot analysis of the lysates using the indicated antibodies was performed.

Supplementary Figure S3

GRK2

a **Antibody Santa Cruz Biotechnology (sc-13143) raised against amino acids 468-689 of human GRK2**

```
huGRK2        468 pplipprgevnaadafdigsfdeedtkgiklldsdqelyrnfpltiserwqqevaetvfdtinaetdrlearkkaknkqlgheedyalgkdcimhgymskmgnpfltqwqrryfylfpnr
muGRK2-1      468 pplipprgevnaadafdigsfdeedtkgiklldsdqelyrnfpltiserwqqevaetvfdtinaetdrlearkkaknkqlgheedyalgkdcivhgymskmgnpfltqwqrryfylfpnr
ratGRK2       468 pplipprgevnaadafdigsfdeedtkgiklldsdqelyrnfpltiserwqqevaetvfdtinaetdrlearkkaknkqlgheedyalgkdcimhgymskmgnpfltqwqrryfylfpnr
hamGRK2-x2    468 pplipprgevnaadafdigsfdeedtkgiklldsdqelyrnfpltiserwqqevaetvfdtinaetdrlearkkaknkqlgheedyalgkdcimhgymskmgnpfltqwqrryfylfpnr
monGRK2       468 pplipprgevnaadafdigsfdeedtkgiklldsdqelyrnfpltiserwqqevaetvfdtinaetdrlearkkaknkqlgheedyalgkdcimhgymskmgnpfltqwqrryfylfpnr


huGRK2        588 lewrgegeapqsllttmeeiqsveetqikerkclllkirggkqfilqcdsdpelvqwkkelrdayreaqqlvqrvpkmknkprspvvelskvplvqrgsangl
muGRK2-1      588 lewrgegeapqsllttmeeiqsveetqikerkclllkirggkqfvlqcdsdpelvqwkkelrdayreaqqlvqrvpkmknkprspvvelskvpliqrgsangl    98 % identity to huGRK2
ratGRK2       588 lewrgedeapqsllttmeeiqsveetqikerkclllkirggkqfvlqcdsdpelvqwkkelrdayreaqqlvqrvpkmknkprspvvelskvpliqrgsangl    98 % identity to huGRK2
hamGRK2-x2    588 lewrgegeapqsllttmeeiqsveetqikerkclllkirggkqfvlqcdsdpelvqwkkelrdayreaqqlvqrvpkmknkprspvvelskvpliqrgsangl    99 % identity to huGRK2
monGRK2       588 lewrgegeapqsllttmeeiqsveetqikerkclllkirggkqfvlqcdsdpelvqwkkelrdayreaqqlvqrvpkmknkprspvvelskvplvqrgsangl    99 % identity to huGRK2
```

b **Antibody Cell Signaling Technology (#3982) raised against amino-terminal residues of human GRK2**

```
huGRK2        1 madleavladvsylmamekskatpaaraskkillpepsirsvmqkyledrgevtfekifsqklgyllfrdfclnhleearplvefyeeikkyekleteeervarsreifdsyimkellac
muGRK2-1      1 madleavladvsylmamekskatpaaraskkillpepsirsvmqkyledrgevtfekifsqklgyllfrdfclnhleeakplvefyeeikkyekleteeervvrsreifdsyimkellac 98 %
ratGRK2       1 madleavladvsylmamekskatpaaraskkillpepsirsvmqkyledrgevtfekifsqklgyllfrdfylnhleeakplvefyeeiekyekleteeervvrsreifdsyimkellac 96 %
hamGRK2-x2    1 madleavladvsylmamekskatpaaraskkillpepsirsvmqkylgdrgevtfekifsqklgyllfrdfclnhleeakplvefyeeikkyekleteeervvrsreifdsyimkellac 97 %
monGRK2       1 madleavladvsylmamekskatpaaraskkillpepsirsvmqkyledrgevtfekifsqklgyllfrdfclnhleearplvefyeeikkyekleteeervarsreifdsyimkellac 100 %
```

Supplementary Figure S3: Alignment of human, mouse, rat, hamster, and monkey GRK2 protein sequences from amino acid 468 to 689 (**a**) and 1 to 120 (**b**). Differing amino acids are highlighted in yellow. Total identity is denoted in per cent.

Supplementary Figure S4

GRK3

Antibody Cell Signaling Technology (#80362) raised against amino acids surrounding lysine 454 (red)

```
huGRK3-1      400 heidrmtltvnvelpdtfspelksllegllqrdvskrlgchgggsqevkehsffkgvdwqhvylqkyppplipprgevnaadafdigsfdeedtkgiklld
muGRK3-1      400 heidrmtltvnvqlpdafspelrsllegllqrdvsqrlgcggggarelkehiffkgidwqhvylrkyppplipprgevnaadafdigsfdeedtkgiklld 89 %
ratGRK3       400 heidrmtltvnvqlpdafspelrsllegllqrdvsqrlgccgggarelkehiffkgidwqyvylrkyppplipprgevnaadafdigsfdeedtkgiklld 88 %
hamGRK3-x3    400 heidrmtltvnvqlpdafspelrsllegllrrdvsqrlgccgggaqevkehiffkgidwqhvylrkyppplipprgevnaadafdigsfdeedtkgiklld 90 %
monGRK3-x1    400 heidrmtltvnvelpdtfspelksllegllqrdvskrlgchgggsqevkehsffkgvdwqhvylqkyppplipprgevnaadafdigsfdeedtkgiklld 100 %
```

Supplementary Figure S4: Alignment of human, mouse, rat, hamster, and monkey GRK3 protein sequences from amino acid 400 to 100. Differing amino acids are highlighted in yellow. Total identity is denoted in per cent.

Supplementary Figure S5

GRK5

Antibody Santa Cruz Biotechnology (sc-518005) raised against amino acids 94-157 of human GRK5

```
huGRK5        94 pdeklgekgkeimtkyltpkspvfiaqvgqdlvsqteekllqkpckelfsacaqsvheylrgep
muGRK5        94 pdenlgakgkeimtkyltpkspvfiaqvgqdlvsqtekkllqspckelfsacaqsvhdylkgdp 89 %
ratGRK5       94 pdenlgakgkeimtkylspkspvfiaqvgqdlvsqtekkllqspckelfsacaqsvhdylkgdp 87 %
hamGRK5-3     94 pdenlgekgkeiitkyltpkspvfiaqvgqdlvsqteakllqrpckelfsacaqsvhdylkgdp 89 %
monGRK5       94 pdeklgekgkeimskyltpkspvfiaqvgrdlvseteekllqkpckelfsacaqsvheylrgep 95 %
```

Supplementary Figure S5: Alignment of human, mouse, rat, hamster, and monkey GRK5 protein sequences from amino acid 94 to 157. Differing amino acids are highlighted in yellow. Total identity is denoted in per cent.

Supplementary Figure S6

GRK6

a **Antibody Cell Signaling Technology (#5878) raised against amino acids surrounding glutamic acid 69 (red) for GRK6**

```
huGRK6-1       30 wrqmlqfphisqceelrlslerdyhslcerqpigrllfrefcatrpelsrcvafldgvaeyevtpddkrkacgrqltqnfl
muGRK6-3       30 wrqmlqfphisqceelrlslerdyhslcerqpigrllfrefcatrpeltrctafldgvseyevtpdekrkacgrrlmqnfl 92 %
ratGRK6-3      30 wrqmlqfphisqceelrlslerdyhslcerqpigrllfrefcatrpeltrctafldgvaeyevtpdekrkacgrrlmqnfl 93 %
hamGRK6-x1     30 wrqmlqfphisqceelrlslerdyhslcerqpigrllfrefcatrpeltrctafldgvaeyevtpdekrkacgrrlmqnfl 93 %
monGRK6-x1     30 wrqmlqfphisqceelrlslerdyhslcerqpigrllfrefcatrpelsrciafldgvaeyevtpdekrkacgrqlmqnfl 96 %
```

b **Antibody Boster Biological Technology (PB9709) raised against amino acids 382-417 of human GRK6**

```
huGRK6-1       382 qspfqqrkkkikreeverlvkevpeeyserfspqar
muGRK6-3       382 qspfqqrkkkikreeverlvkevaeeytdrfssqar 88 %
ratGRK6-3      382 qspfqqrkkkikreeverlvkevaeeytdrfspqar 91 %
hamGRK6-x1     382 qspfqqrkkkikreeverlvkevpeeysehfspqar 97 %
monGRK6-x1     382 qspfqqrkkkikreeverlvkevpeeyserfspqar 100 %
```

Supplementary Figure S6: Alignment of human, mouse, rat, hamster, and monkey GRK6 protein sequences from amino acid 30 to 110 (**a**) and 382 to 417 (**b**). Differing amino acids are highlighted in yellow. Total identity is denoted in per cent.

Supplementary Table S1

Last edited on 19th of october 2021

Overview of mRNA and protein accession identifiers of GRK isoforms in different mammals retrieved from https://www.ncbi.nlm.nih.gov . In case of human, mouse, and rat, the computationally predicted sequences reported are not shown, in case of hamster and monkey, all sequences shown are computationally predicted. Of note, some proteins within one species and GRK isoform are identical despite different accession numbers.

human (homo sapiens)				mouse (mus musculus)				rat (rattus norvegicus)				hamster (cricetulus griseus)				monkey (chlorocebus sabaeus)			
	mRNA accession	protein accession	# AA		mRNA accession	protein accession	# AA		mRNA accession	protein accession	# AA		mRNA accession	protein accession	# AA		mRNA accession	protein accession	# AA
**huGRK2**	NM_001619.5	NP_001610.2	689	**muGRK2-1**	NM_001290818.1	NP_001277747.1	689	**ratGRK2**	NM_012776.2	NP_036908.2	689	**hamGRK2-x1**	XM_027408684.2	XP_027264485.1	699	**monGRK2**	XM_007968626.2	XP_007966817.1	689
				**muGRK2-2**	NM_130863.2	NP_570933.1	647						XM_007650600.4	XP_007648790.2	699				
												**hamGRK2-x2**	XM_027408685.2	XP_027264486.1	689				
													XM_003509943.5	XP_003509991.1	689				
**huGRK3-1**	NM_005160.4	NP_005151.2	688	**muGRK3-1**	NM_177078.4	NP_796052.2	688	**ratGRK3**	NM_012897.3	NP_037029.2	688	**hamGRK3-x1**	XM_007647887.3	XP_007646077.1	401	**monGRK3-x1**	XM_007975215.2	XP_007973406.2	688
**huGRK3-2**	NM_001362778.2	NP_001349707.1	575									**hamGRK3-x2**	XM_007647223.3	XP_007645413.3	245	**monGRK3-x2**	XM_038006312.1	XP_037862240.1	575
				**muGRK3-3**	NM_001285806.1	NP_001272735.1	581					**hamGRK3-x3**	XM_027415948.2	XP_027271749.1	688	**monGRK3-x3**	XM_038006313.1	XP_037862241.1	575
**huGRK5**	NM_005308.3	NP_005299.1	590	**muGRK5**	NM_018869.3	NP_061357.3	590	**ratGRK5**	NM_030829.2	NP_110456.2	590	**hamGRK5-x1**	XM_035436880.1	XP_035292771.1	601	**monGRK5**	XM_007964246.2	XP_007962437.2	598
												**hamGRK5-x2**	XM_035441693.1	XP_035297584.1	601				
												**hamGRK5-x3**	XM_027406842.2	XP_027262643.1	590				
												**hamGRK5-x4**	XM_035441694.1	XP_035297585.1	569				
												**hamGRK5-x5**	XM_027406846.2	XP_027262647.1	426				
**huGRK6-1**	NM_001004106.3	NP_001004106.1	576	**muGRK6-1**	NM_001038018.5	NP_001033107.1	589	**ratGRK6-1**	NM_031657.4	NP_113845.2	589	**hamGRK6-x1**	XM_007623236.4	XP_007621426.2	576	**monGRK6-x1**	XM_008015430.2	XP_008013621.1	576
**huGRK6-2**	NM_002082.4	NP_002073.2	589	**muGRK6-2**	NM_011938.5	NP_036068.2	560	**ratGRK6-2**	NM_001112713.1	NP_001106184.1	560	**hamGRK6-x2**	XM_007623241.4	XP_007621431.2	560	**monGRK6-x2**	XM_008015431.2	XP_008013622.1	560
**huGRK6-3**	NM_001004105.3	NP_001004105.1	560	**muGRK6-3**	NM_001112711.2	NP_001106182.1	576	**ratGRK6-3**	NM_001112712.2	NP_001106183.1	576	**hamGRK6-x3**	XM_007623249.4	XP_007621439.2	481	**monGRK6-x3**	XM_008015433.2	XP_008013624.1	559
**huGRK6-4**	NM_001364164.2	NP_001351093.1	366	**muGRK6-4**	NM_001286063.2	NP_001272992.1	557					**hamGRK6-x4**	XM_027409791.2	XP_027265592.1	576				
				**muGRK6-5**	NM_001286064.2	NP_001272993.1	584					**hamGRK6-x5**	XM_027409792.2	XP_027265593.1	560				
				**muGRK6-6**	NM_001286065.1	NP_001272994.1	542					**hamGRK6-x6**	XM_027409793.2	XP_027265594.1	484				
				**muGRK6-7**	NM_001286066.2	NP_001272995.1	295					**hamGRK6-x7**	XM_027409794.2	XP_027265595.1	481				
				**muGRK6-8**	NM_001377076.1	NP_001364005.1	571					**hamGRK6-x8**	XM_027409795.2	XP_027265596.1	324				
				**muGRK6-9**	NM_001377077.1	NP_001364006.1	311												
